# Prognostic Factors and Optimal Empiric Therapy for Extended-Spectrum Beta-Lactamase (ESBL)-Producing Enterobacterales Bacteremia

**DOI:** 10.7759/cureus.97980

**Published:** 2025-11-27

**Authors:** Tadashi Maeda, Sohei Harada, Dai Nakase, Yusuke Nakamura, Takeo Mori, Katsuhito Kashiwagi, Takahiro Sato, Taito Miyazaki, Yosuke Sasaki

**Affiliations:** 1 Department of General Medicine and Emergency Care, Toho University School of Medicine, Tokyo, JPN; 2 Department of Microbiology and Infectious Diseases, Toho University School of Medicine, Tokyo, JPN

**Keywords:** bacteremia, empiric antimicrobial therapy, esbl-producing enterobacterales, prognostic factors, qsofa score

## Abstract

Background

Bloodstream infections (BSIs) caused by extended-spectrum β-lactamase-producingEnterobacterales (ESBL-E) are associated with increased morbidity and mortality, yet the optimal empiric treatment strategy remains uncertain. We aimed to identify prognostic factors for early clinical response and 30-day mortality in patients with ESBL-E bacteremia and to evaluate the impact of the appropriateness of empiric antimicrobial therapy.

Methods

We conducted a retrospective cohort study at a 916-bed tertiary care hospital in Tokyo, Japan, including all patients with ESBL-E bacteremia between April 2018 and March 2023. Clinical, microbiological, and therapeutic data were extracted from electronic medical records. Independent variables were selected based on prior literature, including age, sex, Charlson Comorbidity Index (CCI), quick sequential organ failure assessment (qSOFA) score, infection setting, invasive device use, C-reactive protein (CRP) level, and appropriateness of empiric antimicrobial therapy. Logistic regression was used to identify predictors of clinical improvement within 72 hours and 30-day all-cause mortality.

Results

A total of 138 patients were included, with a median age of 72.7 years; 83% of infections were caused by *Escherichia coli*. The urinary tract was the most common source of infection (58%). Approximately half of the infections were hospital-acquired (49%), and 46% were community-acquired. Appropriate empiric therapy, defined according to Clinical and Laboratory Standards Institute (CLSI) criteria, was administered in 60% of cases.

Logistic regression analysis revealed that a qSOFA score ≥2 was significantly associated with reduced clinical improvement at 72 hours (odds ratio (OR), 0.29; 95% confidence interval (CI), 0.11-0.76) and with increased 30-day mortality (OR, 9.42; 95%CI, 2.27-39.11). Conversely, community-acquired infection was independently associated with reduced mortality risk (OR, 0.25; 95%CI, 0.06-0.94). Appropriateness of empiric antimicrobial therapy was not significantly associated with either early clinical response or 30-day mortality in multivariable analysis. In subgroup analysis of patients with a qSOFA score ≥2, inappropriate empiric therapy tended to be associated with higher 30-day mortality (OR, 7.22; 95%CI, 0.74-107.88), though this did not reach statistical significance.

Conclusion

Among patients with ESBL-E bacteremia, a qSOFA score ≥2 at the time of blood culture collection was the strongest independent predictor of both poor early clinical response and higher 30-day mortality, regardless of empiric antimicrobial appropriateness. These findings suggest that patient severity at presentation may outweigh the impact of initial antimicrobial selection in determining outcomes. However, potential for clinical deterioration with inappropriate therapy in high-risk patients underscores the need for timely, effective empiric coverage in those with suspected sepsis and elevated qSOFA scores. Risk stratification using qSOFA may support tailored empiric therapy decisions, helping to balance effective patient care with antimicrobial stewardship. Prospective, multicenter studies are warranted to validate these results and refine treatment strategies in the era of increasing antimicrobial resistance.

## Introduction

Extended-spectrum β-lactamases (ESBLs) are enzymes that confer resistance by hydrolyzing penicillins, aztreonam, and cephalosporins. In Japan, ESBL-producing Enterobacterales (ESBL-E) have been steadily increasing since the 2000s, with *Escherichia coli, *particularly CTX-M producers, being the predominant species [1]. Infections caused by ESBL-producing organisms are generally associated with worse clinical outcomes compared to those caused by non-ESBL producers [2]. Moreover, ESBL-E are no longer confined to healthcare settings; they are now widely detected in the community, especially among *E. coli* isolates. According to the Japan Nosocomial Infections Surveillance (JANIS) under the Ministry of Health, Labour and Welfare (MHLW), approximately 20% of *E. coli* isolates exhibit resistance to third-generation cephalosporins, suggesting the production of ESBLs [3]. These findings underscore the critical importance of appropriate antimicrobial stewardship.

Given the potential severity of bloodstream infections (BSIs) caused by ESBL-E, empiric therapy often involves carbapenems as the first-line agents [4]. However, determining which patients require empiric coverage for ESBL-E remains a clinical challenge, particularly as ESBL-E BSIs are increasingly observed in community-acquired cases. Additionally, some patients with ESBL-E infections demonstrate clinical improvement despite receiving empiric therapy with agents to which the organism is not susceptible, further complicating treatment decisions [5].

To address these uncertainties, we conducted a retrospective cohort study of patients with ESBL-E bacteremia in Japan. Our objectives were to characterize the clinical and microbiological features of affected patients and to identify risk factors, including appropriateness of initial antimicrobial therapy, associated with early clinical response (within 72 hours of hospitalization) and 30-day mortality.

## Materials and methods

This study was conducted at a single-center, regional core hospital, Toho University Omori Medical Center, in Tokyo, Japan. The study protocol was carried out in accordance with the principles of Good Clinical Practice and the Declaration of Helsinki, and it was approved by the Institutional Ethics Committee of Toho University School of Medicine (approval number: 22255). Patients and members of the public were not involved in the design, conduct, reporting, or dissemination of this study. They were not consulted to interpret the results or contribute to the writing or editing of this manuscript.

Study participants

All patients with bacteremia caused by ESBL-E were identified through the laboratory information system of Toho University Omori Medical Center, a 916-bed tertiary care hospital located in urban Tokyo. A total of 138 cases were included. The inclusion period spanned 60 months, from April 1, 2018, to March 31, 2023.

Data collection

For each patient, only the first episode of ESBL-E bacteremia during the study period was included. Clinical and laboratory data were retrospectively collected from electronic medical records. These data included age, sex, diagnosis, pathogen, infection setting (community- or hospital-acquired, healthcare-associated), empirical and definitive antimicrobial therapy, comorbid conditions, presence of invasive devices, Charlson Comorbidity Index (CCI) [6], quick Sequential Organ Failure Assessment (qSOFA) score [7], bacteriological resolution, and clinical outcome.

Classification and definitions

Cases were classified as community-acquired, healthcare-associated, or hospital-acquired according to previously established criteria. Community-acquired infection was defined as an infection present or incubating at the time of hospital admission, without any history of recent healthcare exposure. Healthcare-associated infection was defined as an infection present at admission or within 48 hours after admission in patients who had any of the following: residence in a nursing home or long-term care facility, or chronic dialysis within 30 days. Hospital-acquired (nosocomial) infection was defined as an infection that developed ≥48 hours after hospital admission [8]. Based on previous literature, a CCI cutoff value of 3 was used to stratify comorbidity burden [9]. In this study, CCI scores ranged from 0 to 9, with a median of 2 and a mean of 2.9. Therefore, patients were categorized into two groups for analysis: those with high CCI scores (≥3) and those with low CCI scores (<3). Three patients died within 72 hours of admission, the period defined for the initial evaluation, and were therefore classified as not improved.

The onset of bacteremia was defined as the date on which the first blood culture yielding an ESBL-producing organism was obtained. Empirical antimicrobial therapy was defined as the administration of antibiotics before the results of blood cultures were known, whereas definitive antimicrobial therapy was defined as the use of antibiotics after identification of the causative organism and its susceptibility profile.

Bacteremia was classified as hospital-acquired if the culture was collected ≥48 hours after hospital admission. On the other hand, bacteremia was classified as community-acquired if it was present on admission or developed within 48 hours of hospitalization. The appropriateness of empiric therapy with non-β-lactam antibiotics and carbapenems, β-lactam/β-lactamase inhibitor such as piperacillin/tazobactam (PIPC/TAZ) and ampicillin/sulbactam (AMPC/SBT), and cefmetazole (CMZ) was assessed based on in vitro susceptibility results, interpreted according to the Clinical and Laboratory Standards Institute (CLSI) for Antimicrobial Susceptibility Testing M100, 27th edition guidelines [10]. However, penicillins and cephalosporins (excluding cephamycins) without β-lactamase inhibitors, as well as aztreonam, were considered inappropriate regardless of their minimum inhibitory concentrations (MIC).

Microbiological methods

The BD BACTEC FX system (Becton, Dickinson and Company, Franklin Lakes, New Jersey, United States) was used for blood culture processing. ESBL production was determined in accordance with CLSI for Antimicrobial Susceptibility Testing M100, 27th edition guidelines [10].

Identification and antimicrobial susceptibility testing were performed using the WalkAway automated system (Beckman Coulter, Brea, California, United States). ESBL production was confirmed by observing a ≥3 mm increase in zone diameter in the presence of clavulanic acid (CVA), indicating synergistic inhibition.

Investigation of prognostic factors

To identify factors associated with clinical efficacy at 72 hours and all-cause mortality within 30 days of admission, multivariable logistic regression analysis was performed. Independent variables included: appropriateness of empiric antimicrobial therapy (appropriate vs. inappropriate), age, sex, CCI, quick sequential organ failure assessment (qSOFA) score, C-reactive protein (CRP) level, infection setting (community- vs. hospital-acquired), and the presence of invasive devices. Independent variables were selected based on previous studies [9].

Clinical efficacy at 72 hours was defined as the presence of ≥2 of the following four criteria, adapted from previous studies [11]: (i) Resolution of fever (body temperature ≤37.5°C), (ii) Normalization or clear improvement toward normalization of leukocyte count and CRP levels (Regarding leukocyte count and CRP levels: Cases in which a clear trend toward normal values ​​was confirmed through multiple blood tests were included), (iii) Documentation in the medical record indicating a clear clinical improvement as assessed by the attending physician, and (iv) A blood culture specimen drawn within 72 hours after the diagnosis that subsequently became negative.

As a subgroup analysis, outcomes were evaluated in 26 patients with a qSOFA score ≥2. We compared whether there were significant differences in clinical improvement at 72 hours and 30-day mortality between patients who received appropriate empirical antimicrobial therapy and those who did not.

Data analysis

Comparisons between the two groups were conducted using the chi-square test and Fisher’s exact test. A p-value <0.05 was considered statistically significant. All statistical analyses were performed using R (The R Foundation for Statistical Computing, Vienna, Austria) and EZR [12], a graphical user interface for R designed for medical statistics.

## Results

During the study period, 138 patients experienced an episode of ESBL-E bacteremia. The annual number of ESBL-E bacteremia cases was approximately 30, with no notable increasing or decreasing trend observed over the five-year period. The most common causative organism was *E. coli*, isolated in 115 cases (83%). Other pathogens included *Klebsiella pneumoniae*, *Proteus mirabilis*, and *Klebsiella oxytoca*. The most frequent source of infection was urinary tract infection (n = 80, 58%), followed by cholecystitis/ cholangitis, primary bloodstream infections, pneumonia, vascular catheter infection and intra-abdominal infection (Table 1).

**Table 1:** Annual case counts for bacteremia pathogens and bacteremia sources (N=138)

Parameters		Frequency (Percentage)
Annual patients	2018	31 (22%)
	2019	25 (18%)
	2020	25 (18%)
	2021	28 (20%)
	2022	29 (21%)
Isolated species in cases	Escherichia coli	115 (83%)
	Klebsiella pneumoniae	21 (15%)
	Proteus mirabilis	1 (0.7%)
	Klebsiella oxytoca	1 (0.7%)
Bacteremia source	Urinary tract	80 (58%)
	Cholecystitis,cholangitis	25 (18%)
	Primary bloodstream infections	14 (10%)
	Pneumonia	7 (5.1%)
	Vascular catheter infection	5 (3.6%)
	Intra-abdominal infection	3 (2.2%)
	Other	4 (2.9%)
Empirical antimicrobiotics prescribed	Meropenem	48 (35%)
	Piperacillin/tazobactam	26 (19%)
	Cefmetazole	8 (5.8%)
	Sulbactam/ampicillin	8 (5.8%)
	Levofloxacin	2 (1.4%)
	Cefepime	12 (8.7%)
	Ceftriaxone	24 (17%)
	Other	10 (7.2%)

The median age of patients was 72.7 years. There were 68 hospital-acquired cases (49%), 64 community-acquired cases (46%), and six healthcare-associated cases (4%). Empiric and targeted antibiotic therapy were distinguished if administered before or after the result of the antimicrobial susceptibility test. Appropriate antibiotic therapy was determined based on the results of antibiotic susceptibility testing. Appropriate empiric antimicrobial therapy was administered in 83 cases (60%). Empirical antimicrobial therapy at the start of treatment included meropenem in 48 cases, piperacillin/tazobactam in 26 cases (one case: intermediate and otherwise susceptible), cefmetazole in eight cases (all susceptible), sulbactam/ampicillin in eight cases (susceptible in one case and resistant in the remainder), levofloxacin in two cases (both resistant), cefepime in 12 cases, and ceftriaxone in 24 cases. Other agents used (one case each) included ampicillin, piperacillin, cefazolin, cefotiam, cefotaxime, and vancomycin (Table 1). Fewer than 20% of patients (n = 26) had a qSOFA score ≥2 at presentation. Detailed patient characteristics are summarized in Table 2.

**Table 2:** Clinical background *Devices include urinary catheters, indwelling vascular catheters, and other indwelling catheters. Comparisons between the two groups were conducted using the chi-square test. A P value of <0.05 was considered statistically significant. qSOFA: quick sequential organ failure assessment; CRP: C-reactive protein

Parameters	Appropriate empiric antimicrobial therapy (n=83), n (%)	Inappropriate empiric antimicrobial therapy (n=55), n (%)	P-value	χ²
Charlson comorbidity index			0.119	5.862
0	14 (17%)	7 (13%)		
1〜2	31 (37%)	20 (36%)		
3〜4	15 (18%)	19 (35%)		
≥5	23 (28%)	9 (16%)		
With Medical Devices*	34 (41%)	20 (36%)	0.559	0.132
qSOFA at the time of admission≥2	20 (24%)	6 (11%)	0.074	2.949
CRP≥10mg/dL	47 (57%)	35 (64%)	0.48	0.415
Community-acquired infection	40 (48%)	24 (44%)	0.607	0.123
Age ≥75 years	49 (59%)	36 (66%)	0.479	0.337
Male sex	44 (53%)	30 (55%)	1	0

Logistic regression analysis revealed that a qSOFA score ≥2 was significantly associated with reduced clinical improvement at 72 hours (OR, 0.29; 95%CI, 0.11-0.76). In terms of 30-day mortality, patients with a qSOFA score ≥2 had a significantly higher risk of death (OR 9.42, 95%CI 2.27-39.11), whereas community-acquired infections were associated with a significantly lower mortality rate (OR 0.25, 95%CI 0.06-0.94) (Tables 3, 4).

**Table 3:** Factors affecting efficacy after 72 hours (N=138) The table presents the results obtained from logistic regression analysis. A P value of <0.05 was considered statistically significant. Patients with qSOFA ≥ 2 at admission were significantly less likely to have improved after 72 hours(P=0.0117). qSOFA: quick sequential organ failure assessment; CRP: C-reactive protein

Parameters	Odds ratio	95% CI	P-value
Appropriate Antibiotics Group	1.86	0.87 – 3.98	0.1116
Charlson Index ≥ 3	0.94	0.80 – 1.10	0.4361
Admission qSOFA ≥ 2	0.29	0.11 - 0.76	0.0117
CRP ≥ 10mg/dL	1.41	0.66 – 3.02	0.3764
Community-Acquired Infection	1.08	0.51 – 2.27	0.8495
Age ≥ 75 years	1.25	0.59 – 2.65	0.5634
Male sex	1.85	0.88 – 3.91	0.1049

**Table 4:** Factors affecting mortality after 30 days of hospitalization (N=138) The table presents the results obtained from logistic regression analysis. A P value of <0.05 was considered statistically significant. Admission qSOFA≥2 has significantly higher mortality (p=0.002), and community-acquired infections have significantly lower mortality (p=0.0359). qSOFA: quick sequential organ failure assessment; CRP: C-reactive protein

Parameters	Odds ratio	95% CI	P-value
Appropriate Antibiotics Group	0.49	0.13 – 1.90	0.3003
Charlson Index ≥ 3	0.97	0.69 – 1.38	0.8787
Admission qSOFA ≥ 2	9.42	2.27 – 39.11	0.002
CRP ≥ 10 mg/dL	1.45	0.34 – 6.19	0.6198
Community-Acquired infection	0.25	0.06 – 0.94	0.0359
Age ≥ 75 years	2.15	0.51 – 9.04	0.2946
Male sex	1.05	0.29 – 3.84	0.9381

Appropriate empirical antimicrobial therapy was not a significant factor in either analysis. Subsequently, 26 patients with a qSOFA score ≥2, identified as an independent predictor of both clinical improvement at 72 hours and 30-day mortality, were stratified into two groups according to the appropriateness of initial empiric antimicrobial therapy. Although not statistically significant (p = 0.051), there was a numerical trend toward higher 30-day mortality in patients who received inappropriate initial antimicrobial therapy (OR, 7.22, 95%CI 0.74-107.88) (Table 5).

**Table 5:** Evaluation of the appropriateness of empirical antimicrobial therapy in cases with a qSOFA score≥2 (N=26) Comparisons between the two groups were conducted using Fisher’s exact test. A P value of <0.05 was considered statistically significant. qSOFA: quick sequential organ failure assessment

Parameters	Appropriate treatment (N=20)	Inappropriate treatment (N=6)	p-value	OR	95%CI
Clinical effectiveness at 72 hours					
Improved	8	2	1	0.76	0.06-6.89
Not improved	12	4			
Outcome at 30 days					
Death	4	4	0.051	7.22	0.74-107.88
Survival	16	2			

## Discussion

Bloodstream infections caused by ESBL-E have been reported to be associated with significantly higher mortality rates compared to those caused by non-ESBL-E organisms [13]. When ESBL-E involvement is suspected, empiric therapy typically involves the use of carbapenem antibiotics [4]. In addition, several clinical factors-including advanced age, underlying comorbidities, and inappropriate initial antimicrobial therapy-have been shown to contribute to mortality, particularly in critically ill patients [14,15].

For clinicians, inappropriate empiric antimicrobial therapy is one of the most concerning consequences of the increasing incidence of ESBL-E infections. However, in the present study, initial inappropriate therapy was not associated with increased 30-day mortality in multivariable analysis. Instead, a qSOFA score ≥2 at the time of blood culture collection emerged as an independent predictor of both failure to improve within 72 hours and 30-day mortality, regardless of the appropriateness of empiric therapy or other known risk factors such as CCI, age, sex, elevated CRP, hospital-acquired infection, and device implantation.

Although inappropriate empiric antibiotic selection is a justifiable concern for clinicians, these findings suggest that the patient’s initial clinical condition plays a more critical role in determining outcomes. Nonetheless, it is noteworthy that among patients with a qSOFA score ≥2, those who received inappropriate initial antimicrobial therapy tended to have a higher 30-day mortality rate. Although this difference did not reach statistical significance, likely due to the small sample size, there remains a concern that inappropriate therapy may adversely impact prognosis in patients with suspected sepsis and elevated qSOFA scores at the time of blood culture collection. To clarify this relationship, a multicenter study with a larger cohort of patients having higher qSOFA scores is warranted. The predictive factors identified in this study may assist clinicians in determining which patients are most likely to benefit from empirical carbapenem therapy or other appropriate antimicrobial agents. Given the global increase in multidrug-resistant organisms, including ESBL-producing bacteria [16], such risk stratification can aid in the optimization of antimicrobial stewardship and help prevent the emergence of further resistance, particularly carbapenem-resistant Enterobacterales.

Although qSOFA has been criticized for its limited sensitivity in diagnosing sepsis and predicting hospital mortality [17,18], our findings indicate that it may still have value as a pragmatic prognostic tool in the context of ESBL-E bacteremia. Its simplicity and bedside applicability make it particularly attractive in resource-limited settings, where rapid risk stratification can guide early management decisions. In particular, patients with ESBL-E bacteremia and a qSOFA score ≥2 had poorer outcomes and may require more vigilant management, including timely administration of appropriate empiric antimicrobials. Another important finding was the relatively low 30-day mortality rate observed in patients with community-acquired ESBL-E bacteremia. These results suggest that empiric carbapenem therapy targeting ESBL-producing organisms may not be necessary for patients with community-onset infections who have a qSOFA score ≥ 2, thereby helping to avoid overuse of broad-spectrum agents and reducing selective pressure for carbapenem-resistant Enterobacterales.

Several limitations of this study should be acknowledged. First, due to its retrospective nature, detailed information such as the exact duration and number of previous antibiotic exposures could not be obtained for all patients. Second, the study was conducted at a single university hospital in Japan, and the distribution of ESBL genotypes may differ across geographic regions, thereby limiting generalizability [19,20]. Third, the majority of the ESBL-producing isolates were* E. coli*; therefore, the findings may not be applicable to settings in which other ESBL-producing organisms, such as *K. pneumoniae*, predominate [21].

## Conclusions

This study identified an admission qSOFA score ≥2 as a strong independent predictor of early clinical response and 30-day mortality in patients with ESBL-E bacteremia. These results emphasize the importance of assessing patient severity at presentation and suggest that risk-stratified empirical therapy may support both improved patient outcomes and responsible antimicrobial stewardship. Future prospective, multicenter studies are needed to validate these findings and further refine clinical strategies for managing ESBL-E bacteremia in an era of rising antimicrobial resistance.
